# Prof. C. A. Lee

**Published:** 1849-11

**Authors:** 


*Prof. C. A. Lee.*—We have neglected, among other omissions, to state that Prof. Lee sailed for Europe in June last, for relaxation and professional observations. His numerous friends will be gratified to learn that his health has been greatly improved by his travels. He expects to sail for home in the early part of December, and arrive in season to discharge his duties during the approaching session of the Medical College in this place.

